# Subcutaneous immunoglobulin for maintenance treatment in chronic inflammatory demyelinating polyneuropathy (The PATH Study): study protocol for a randomized controlled trial

**DOI:** 10.1186/s13063-016-1466-2

**Published:** 2016-07-25

**Authors:** Ivo N. van Schaik, Nan van Geloven, Vera Bril, Hans-Peter Hartung, Richard A. Lewis, Gen Sobue, John-Philip Lawo, Orell Mielke, David R. Cornblath, Ingemar S. J. Merkies, on behalf of the PATH study group

**Affiliations:** 1 Department of Neurology, Academic Medical Centre, University of Amsterdam, Meibergdreef 9, 1105 AZ Amsterdam, The Netherlands; 2 Department of Biostatistics and Bioinformatics, Leiden University Medical Center, Leiden, The Netherlands; 3 Department of Medicine (Neurology), University Health Network, University of Toronto, Toronto, Canada; 4 Department of Neurology, Heinrich Heine University, Dusseldorf, Germany; 5 Department of Neurology, Cedars-Sinai Medical Center, Los Angeles, CA USA; 6 Department of Neurology, Nagoya University Graduate School of Medicine, Nagoya, Japan; 7 CSL Behring Biotherapies for Life™, Marburg, Germany; 8 Department of Neurology, Johns Hopkins University School of Medicine, Baltimore, MD USA; 9 Department of Neurology, Maastricht University Medical Center, Maastricht, The Netherlands

**Keywords:** CIDP, inflammatory neuropathy, Subcutaneous immunoglobulins, SCIg, intravenous immunoglobulins, IVIg, RCT

## Abstract

**Background:**

Subcutaneous administration of Ig (SCIg) has gained popularity as an alternative route of administration but has never been rigorously examined in chronic inflammatory demyelinating polyneuropathy (CIDP).

**Methods/design:**

The primary objective of the PATH study (Polyneuropathy and Treatment with Hizentra) is to determine the efficacy of two different doses of SCIg IgPro20 (0.2 g/kg bw or 0.4 g/kg bw) in a 24-week maintenance treatment of CIDP in comparison to placebo. The primary efficacy endpoint will be the proportion of patients who show CIDP relapse (1-point deterioration on the adjusted Inflammatory Neuropathy Cause and Treatment (INCAT) disability score) or are withdrawn within 24 weeks after randomization for any reason. IVIg-dependent adult patients with definite or probable CIDP according to the European Federation of Neurological Societies/Peripheral Nerve Society who fulfil the inclusion and exclusion criteria will be eligible. Based on sample-size calculation and relapse assumptions in the three arms, a sample size of 58 is needed per arm (overall sample size will be 350, of which 174 will be randomized). All eligible patients will progress through three study periods: an IgG dependency period (≤12 weeks) to select those who are Ig dependent; an IVIg restabilization period (10 or 13 weeks), which will be performed using the 10 % IgPro10 product; and an SC treatment period (24 weeks, followed by a 1-week completion visit after last follow-up). Patients showing IVIg restabilization will be randomized to demonstrate the efficacy of SCIg IgPro20 maintenance treatment over placebo. After completing the study, subjects are eligible to enter a long-term, open-label, extension study of 1 year or return to their previous treatment. In case of CIDP relapse during the 24-week SC treatment period, IgPro10 rescue medication will be offered. Safety, tolerability, and patients’ preference of Ig administration route will be examined.

**Discussion:**

The PATH trial, which started in March 2012, is expected to finish at the end of 2016. The results will increase knowledge about the efficacy, safety, and tolerability of SCIg in maintenance management of CIDP patients.

**Trial Registration:**

ClinicalTrials.gov, NCT01545076. Registered on 1 March 2012.

## Background

Chronic inflammatory demyelinating polyneuropathy (CIDP) is an acquired neuropathy with an assumed autoimmune-mediated pathogenesis [1]. CIDP runs a progressive, relapsing–remitting, or monophasic course and can lead to significant activity limitations and participation restrictions with a decrement in quality of life expectations [2–4].

An estimated two thirds of patients with CIDP need long-term treatment [5]. In a comparison of safety profiles, IVIg is the preferred long-term maintenance treatment over corticosteroids or plasma exchange in CIDP patients. An alternative route of Ig administration, subcutaneous Ig (SCIg), has been used successfully in patients with immunodeficiency syndromes for more than 25 years [6]. SCIg infusions are well tolerated, efficacious, and preferred by many of these patients [7–10]. Furthermore, this route of administration increases patient compliance, autonomy, and quality of life and leads to cost-savings [11–14]. Similar preference has been suggested in patients with CIDP treated with SCIg.

The development of highly concentrated Ig preparations and special pumps to administer larger volumes subcutaneously has also raised interest in this route of administration for patients with inflammatory neuropathies. Several case series [14–22], a relatively large, prospective, observational study [23] and one small, randomized, controlled trial [24], have reported clinical efficacy and safety of weekly SCIg to treat CIDP. A 1-year, open-label, follow-up study has suggested SCIg may be used as a long-term maintenance treatment in CIDP [25]. However, the efficacy, safety, and tolerability of weekly SCIg in CIDP have not been studied in well-controlled and adequately powered randomized clinical trials. The findings of the PATH trial are expected to increase the knowledge about the use of SCIg in the maintenance management of CIDP patients.

## Methods/design

The PATH trial is a randomized, multicenter, double-blind, placebo-controlled, parallel-group phase III study with three arms, aiming to investigate two different doses of SCIg IgPro20 (Hizentra®, CSL Behring, Bern, Switzerland) for maintenance treatment of patients with CIDP. The study began in March 2012 and is expected to finish at the end of 2016. The trial protocol has been approved by the ethics committees of all participating centers (listed in the Appendix). Protocol amendments are covered in a separate section below. The conduct of the trial was overseen by a steering committee and an independent data monitoring committee; committee members are listed at the end of the manuscript. This study is registered with ClinicalTrials.gov number NCT01545076.

### Primary objective

The primary objective is to determine the efficacy of IgPro20 in the maintenance treatment of CIDP patients.

### Secondary and exploratory objectives

- To determine the efficacy, safety, and tolerability of IgPro20, with additional clinical outcome measures
- To determine the safety and efficacy of IVIg IgPro10 restabilization and rescue therapy
- To determine health-related quality of life (HRQL) following treatment with IgPro20
- To explore additional safety and efficacy endpoints, serum IgG levels, and the effect of IgPro20 on nerve conduction

### Patients

#### Inclusion criteria

Adults (age ≥ 18 years) with definite or probable CIDP according to the EFNS/PNS criteria may enter the trial if they responded to IVIg treatment as assessed by the treating physician within 8 weeks before enrollment [26]. Written informed consent is obtained by the local investigator before entry into the study.

#### Exclusion criteria

1. Any polyneuropathy of other causes, including multifocal motor neuropathy; monoclonal gammopathy of uncertain significance with antimyelin-associated glycoprotein IgM antibodies; hereditary demyelinating neuropathy; polyneuropathy, organomegaly, endocrinopathy, monoclonal protein and skin change syndromes; lumbosacral radiculoplexus neuropathy; polyneuropathy associated with diabetes mellitus; polyneuropathy associated with systemic illnesses; or drug- or toxin-induced polyneuropathy
2. Any other disease that may cause neurological symptoms and signs or that may interfere with treatment or outcome assessments
3. Severe conditions that may interfere with an evaluation of the study product or satisfactory conduct of the study such as current malignancy or history of allogeneic bone marrow/stem cell transplant, cardiac insufficiency (New York Heart Association Classes III/IV), cardiomyopathy, significant cardiac arrhythmia requiring treatment, unstable or advanced ischemic heart disease, congestive heart failure or severe hypertension, chronic kidney disease stage IV and V, known hyperprolinemia, known bleeding disorders, severe skin disease at the planned injection sites, alcohol, drug or medication abuse
4. History of thrombotic episodes within the 2 years before enrollment, such as pulmonary embolism, deep vein thrombosis, myocardial infarction, thromboembolic stroke or known hypercoagulable state
5. Known allergic or other severe reactions to blood products including intolerance to previous IVIg, history of hemolysis after IVIg infusion, aseptic meningitis, recurrent severe headache, hypersensitivity, or severe generalized skin reaction
6. Treatment with the following:
  Within 3 months before enrollment: plasma exchange
  Within 6 months before enrollment: cyclophosphamide, interferon, tumor necrosis factor–alpha inhibitors, fingolimod, or any other immunosuppressive medications
  Within 12 months before enrollment: rituximab or alemtuzumab
  With a change in treatment within 3 months before enrollment: methotrexate, azathioprine, or mycophenolate; patients on corticosteroids not on a maintenance dose (usually below 20 mg/day prednisone equivalent) and where the dosage is likely to be tapered during the duration of the trial; or patients requiring more than 1.6 g/kg IgG every 4 weeks
7. Patients with the following laboratory results:
  Serum IgA level less than 5 % of the lower limit of normal
  Positive result at screening on any of the following viral markers: human immunodeficiency virus-1 or 2, or hepatitis B or C virus
  Abnormal laboratory parameters: creatinine greater than 1.5 times the upper limit of normal (ULN), blood urea nitrogen greater than three times the ULN if the increase is related to potential kidney disease, or hemoglobin less than 10 g/dL
8. Fulfilling the following general criteria: inability to comply with study procedures and treatment regimen; mental condition rendering the patient unable to understand the nature, scope, and possible consequences of the study; pregnancy or nursing mother; intention to become pregnant during the course of the study; female patients of childbearing potential either not using or not willing to use a medically reliable method of contraception for the entire duration of the study or not sexually abstinent for the entire duration of the study or not surgically sterile; participation in another clinical study or use of another investigational medicinal product within 3 months before enrollment; employee at the study site; or spouse/partner or relative of any study staff

### Study procedures

**Fig. 1:**
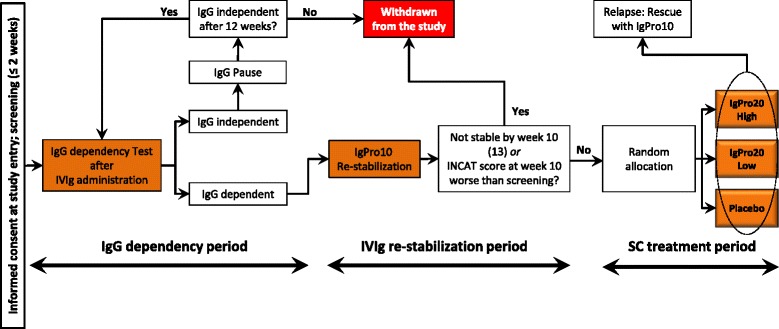
Study design. Diagram explains how patients flow through the different study periods. *IgG* immunoglobulin G, *IVIg* intravenous immunoglobulin, *INCAT* Inflammatory Neuropathy, Cause and Treatment

After being screened, all eligible patients will progress through three study periods: an IgG dependency test period (up to 12 weeks), an IVIg restabilization period (10 or 13 weeks), and an SC treatment period (25 weeks; Fig. 1).

#### IgG dependency test period

In this period, no IgG treatment is given. Patients are monitored by collecting grip strength, Inflammatory-Rasch-built Overall Disability Scale (I-RODS), and INCAT total score data every 2 weeks, alternating by a site visit or by phone. Any patient showing a clinically meaningful deterioration that is confirmed by the investigator at the site will enter the IVIg restabilization period immediately. A clinically meaningful deterioration is defined as a total INCAT disability score increase by ≥ 1 point, I-RODS deterioration by ≥ 4 points (using the centile metric), or a mean grip strength deterioration by ≥ 8 kiloPascal (kPa) in one hand using the handheld vigorimeter [27–30].

Patients will be instructed on how to self-assess grip strength and I-RODS at home as part of a weekly diary. Patients will be performing three assessments for each hand in arbitrary order (with approximately 30 seconds rest between each assessment) on a daily basis and at a fixed time during the day. The mean grip strength for each hand will be used to determine IgG dependency.

Patients who have stable disease or who show improvement will be asked at week 4 to delay the next IVIg administration and to continue with self-assessment of grip strength and I-RODS at home. Patients who do not show signs of IgG dependency after a maximum of 12 weeks will be withdrawn from the study.

#### IVIg restabilization period

In this period, patients will receive IVIg IgPro10 (Privigen®, CSL Behring, Bern, Switzerland) for up to 13 weeks at the study site. The total dose/volume of IgPro10 will be calculated based on the patient’s body weight (bw) with a maximum of 200 g. The loading dose is 2 g/kg (week 1) and 1 g/kg at week 4, 7, and 10 and, if necessary, week 13. The infusion rates are in conformity with the recommended rates for IgPro10 and with the market authorization for IgPro10 (Privigen®) with a maximum of 100 g per infusion day. [31]

Only patients whose INCAT total score improves to at least the INCAT total score recorded at the screening visit (i.e., ≥ INCAT score at screening) and who maintain a stable INCAT total score at weeks 7 and 10 (or at weeks 10 and 13) are eligible for randomization. All other patients will not be randomized and will be withdrawn from the study.

#### SC treatment period

Three-arm randomization will be performed: one group will receive IgPro20 at 0.2 g/kg bw plus placebo to match the volume in all three groups, one group will receive IgPro20 at 0.4 g/kg bw, and one group will receive only placebo. The total dose/volume of IgPro20 will be calculated on the basis of the bw. The weekly SC infusion of IgPro20 or placebo will be performed on 1 or 2 consecutive days in two sessions using special infusion pumps. Multiple injection sites and two pumps may be used depending on the total volume to be administered. The maximum rate is 20 mL/h in week 1, and 35 mL/h for subsequent infusions. The maximum volume per injection site is 20 mL/site in week 1 and 50 mL/site for the subsequent infusions. Patients are advised to change the injection site(s) with each administration.

Patients (or their caregiver) will be trained to apply SC home therapy during the first four SC treatment sessions at the site. If needed, up to four additional trainings will be offered. Patients will also receive detailed written instructions. To ensure compliance, patients are instructed to follow the treatment instructions carefully and to contact the treating physician/study nurse to discuss any problems with SC infusion.

Patients will bring their used, partially used, and unused vials of IgPro20/placebo every visit to the site. Treatment compliance will be monitored through a drug accountability form checking the unique vial numbers used by a patient and the recorded infusion scheme. A completion visit will be performed at SC week 25 for patients who complete the SC treatment period. Patients experiencing a CIDP relapse during the SC treatment period will receive, within 1 week, IgPro10 as IVIg rescue medication (2 g/kg bw) and will undergo a completion visit before the start of the rescue medication. Patients who withdraw for any other reason will undergo the completion visit within a week of discontinuation.

### Randomization and masking

Randomization will be controlled centrally based on a predefined randomization schedule using block randomization using an Interactive Web and Voice Response System (IWRS) system maintained by an external service provider. Randomization was stratified by for regions (Japan/non-Japan). Access to the randomization list is restricted to designated people of the service provider not involved in the conduct or analysis of the trial. The treating physician (see below) transmits data critical for the randomization procedure via the IWRS system. All patients and study personnel will be blinded. Standard measures will be taken for the placebo and IgPro20 to ensure adequate blinding. A “two-physician” approach is implemented to reduce the chance of potential study unblinding. The “treating” physician will be the primary contact for the patient and will be responsible for all patient-related questions, adverse event (AE) evaluation, and for all other study-related tasks. A second “evaluating” physician will be responsible for assessment of efficacy variables. The evaluating physician does not have access to any data collected by the treating physician. For interim analysis and ongoing risk-benefit evaluations, members of the independent data monitoring committee may need to be unblinded. Access to study documents containing information on IgG levels and treatment groups will be restricted to people not directly involved in the study.

In case of an emergency and if necessary to make an adequate treatment decision, the blind can be broken by the treating physician using the interactive voice/web response system. Patients in whom the blind code has been broken will be discontinued from the study after a completion visit.

### Outcome assessment

#### Primary outcome

Primary outcome is defined as the percentage of patients who have a CIDP relapse during SC treatment or who are withdrawn from the study during SC treatment for any reason.

CIDP relapse is defined as a deterioration (i.e., increase) by at least 1 point in the total adjusted INCAT score during the SC treatment period visit compared with baseline. Baseline scores are defined as the scores assessed at the end of the IVIg restabilization period. The INCAT disability scale ranges from 0 (healthy) to 10 (unable to make any purposeful movements with arms or legs) [32].

#### Secondary outcomes

Secondary outcomes are:

1. Between-group differences of the median changes from baseline to completion visits in INCAT score, mean grip strength for both hands separately, Medical Research Council (MRC) sum score, and I-RODS
2. Time to CIDP relapse or withdrawal for any other reason in SC treatment period
3. Time to improvement on IgPro10 restabilization therapy (INCAT, I-RODS, and grip strength)
4. Median changes before and at the end of IgPro10 restabilization or rescue therapy in mean grip strength, MRC sum score, I-RODS, and INCAT disability score
5. Time to improvement after CIDP relapse in the SC treatment period with IgPro10 rescue therapy, defined as a decrease in INCAT score back to or below baseline value

Grip strength will be measured as described above. MRC sum score is determined by assessing bilateral shoulder abduction, elbow flexion, wrist extension, index finger abduction, hip flexion, knee extension, foot dorsiflexion, and great toe dorsiflexion. The MRC sum score is the sum of all 16 muscle scores and ranges from 0 (paralysis) to 80 (normal strength) [33, 34].

The I-RODS is an outcome measure that captures activity and social participation in patients with immune-mediated peripheral neuropathies [28]. The 24-item questionnaire covers a wide range of tasks of daily life that are each to be rated as “impossible to perform,” “able to perform with difficulties,” or “easily performed.” The summed raw score will subsequently be transformed to a centile score that ranges from 0 (most severe activity and social participation limitations) to 100 (no activity and social participation limitations).

#### Assessment schedule of primary and secondary outcome variables

INCAT scores, grip strength, MRC sum score, and I-RODS will be assessed at the screening visit; during the IgG dependency test period as described above, before IVIg infusion at weeks 1, 4, and 7; at baseline (weeks 10 or 13); at all visits during the SC treatment period, including the completion visit; and at any unscheduled visit.

#### Safety outcomes

Patient safety will be monitored throughout the study. AEs per infusion and number and the percent of patients with AEs will be collected for IgPro20 and for IgPro10 separately. AEs, including serious AEs, will be documented at each site visit and additionally for Japan at 4 weeks after last dose. Potential hemolysis will be assessed during the phone call on day 8 of the IVIg restabilization period. Medications will be reviewed at each site visit as well as 4 weeks after the last dose in Japan.

#### Exploratory outcomes

Nerve conduction studies will be performed in a standardized manner after appropriate warming of the extremities. An electrophysiology manual will be provided to all participating centers describing in detail all nerve-conduction-study procedures. Nerve conduction studies will be performed at baseline and at the completion visit.

Quality of life will be assessed using the EuroQoL 5-Dimension Questionnaire (EQ-5D), Treatment Satisfaction Questionnaire for Medication (TSQM), and Work Productivity and Activity Impairment Questionnaire for General Health (WPAI-GH) [35–39]. These instruments will be completed by the patients themselves at screening, baseline, SC week 9, and at the completion visit.

The EQ-5D is a simple, generic measure of health status, consisting of two components: a 0 to 100 mm visual analog scale (VAS) assessing overall health on the day of assessment and five questions covering five health dimensions [39]. The TSQM (version 1.4) is a 14-item general instrument that measures the major dimensions of satisfaction with a medication [36]. Scores on the TSQM scales range from 0 (indicating poor satisfaction) to 100 (indicating perfect satisfaction).

The WPAI-GH questionnaire was developed to measure the effect of general health and symptom severity on work productivity and regular activities [38]. A higher score on the WPAI-GH indicates greater impairment and less productivity.

Patient preference for IV or SC treatment will be assessed via a questionnaire consisting of three options: prefer current treatment [SCIg], prefer previous treatment [IVIg], and have no preference. In addition, a selection of predefined reasons for their preference will be provided. The patient preference for treatment questionnaire will be completed at SC week 9 and at the completion visit. Other exploratory outcome measures are hematology, serum chemistry, virology assessments, vital sign measurements, and physical examination.

Pharmacokinetic variables will be restricted to serum IgG levels collected at the screening visit; before the first IVIg infusions on day 1, week 1, week 4, at baseline, and at SC weeks 9 and 17; and at the completion visit. For patients who receive IVIg rescue medication, IgG levels will additionally be assessed before the first infusion of IVIg rescue medication, before the final infusion of the loading dose, and at each subsequent maintenance dose.

### Statistical analysis

#### Sample size

The study is powered to show that the percentage of patients having a CIDP relapse or withdrawing from the study during the SC treatment will be statistically significantly higher for the placebo group than for at least one of the examined IgPro20 doses arms. A monotonic dose response is expected with placebo ≥ IgPro20 low dose ≥ IgPro20 high dose.

The percentages of patients who reach an endpoint (i.e., relapse or withdraw during SC treatment) will be assumed to be 35 % for the IgPro20 high dose, 52 % for the IgPro20 low dose, and 65 % for placebo. These numbers are based on the (recalculated) data of the ICE study extension period [32], which are adopted based on differences in the study setup. The fraction of IVIg independent patients included is assumed to be 15 %; those patients are believed to have a relapse rate of 10 % regardless of treatment.

The exact Cochran-Armitage trend test with equally spaced scores has been used for the sample-size calculation. With a one-sided significance level of 2.5 %, a sample size of 58 is needed in each treatment arm to achieve a power of 90 % in an intention-to-treat analysis based on the above assumptions. Thus, the overall sample size will be 174 patients treated either with IgPro20 or placebo. Accounting for patients who will not pass the IgG dependency test and IVIg restabilization period, up to 350 patients need to be screened to ensure that 174 patients are treated with IgPro20 or placebo.

#### General considerations

Analyses will be based on the following populations: total set, safety data set (SDS), pre-randomization safety data set (PSDS), intention-to-treat set (ITTS), per protocol set (PPS), and rescue safety data set (RSDS). A blind data review meeting will be arranged to discuss all protocol deviations to decide which patients will be excluded from certain analyses. A reference visit is defined per study period, and this visit is used to assess changes within the period using summary statistics (Table 1). All analyses will be done using SAS® version 9.1.3 (SAS Institute Inc., Cary, NC) or higher.

**Table 1:** Definition of reference visit and last visit of study periods

Period	First visit of period	Reference visit(s) of period	Last visit	
IgG dependency	Screening	Prior to AMD3: INCAT. After AMD3: INCAT: day 14 phone call I-RODS / mean grip strength: peak value within first 4 weeks	Week 1 day 1, before start of IVIg infusion	…or last visit before/at withdrawal
IVIg restabilization	Week 1 day 1 at start of IVIg infusion	Week 1 day 1 at start of IVIg infusion. If not available, last visit of IgG Dependency Period	SC week 1 before start of SC infusion	
SC treatment	SC week 1 at start of SC infusion	Baseline (week 10/13)	Week 25 visit or first IVIg infusion for rescue before IVIg infusion	
IVIg rescue	first IVIg infusion for rescue at start of IVIg infusion	First IVIg infusion for rescue at start of IVIg infusion	Completion visit	

*IgG* immunoglobulin G, *INCAT* Inflammatory Neuropathy Cause and Treatment, *IVIg* intravenous immunoglobulin, *I-RODS* inflammatory-Rasch-built overall disability scale, *SC* subcutaneous

#### Efficacy evaluation primary outcome

The exact Cochran-Armitage trend test will be used for the primary outcome. This comparison will be calculated to test for superiority of at least one IgPro20 dose over placebo at a one-sided type-I error of α = 0.025. If the hypothesized superiority is demonstrated, one-sided Fisher’s exact tests will be used for the subsequent comparisons: placebo vs. low dose IgPro20, and low dose IgPro20 vs. high dose IgPro20.

The proportions and corresponding two-sided 95 % Wilson-Score confidence intervals will be calculated for each treatment group. Point estimates for the difference in proportions and the corresponding exact two-sided 95 % confidence intervals will be calculated for all pair-wise treatment comparisons. The primary endpoint analyses, including all sensitivity analyses (see below), will be performed based on the ITTS and repeated for the PPS.

#### Efficacy evaluation secondary outcomes

An overview of the planned analyses is provided in Table 2. Time to improvement during the IVIg restabilization period will be analyzed separately for INCAT total score (decrease by ≥ 1 point), mean grip strength (increase by ≥ 8 kPa), dominant/nondominant), and I-RODS (increase by ≥ 4 points), as well as overall using Kaplan-Meier estimates. For the overall analysis, if a subject has multiple dates of improvement, the first will be used. Subjects who do not record an improvement will be censored at the date of their last visit. Analyses will be based on the PSDS. Time to improvement after relapse in the SC treatment period will be analyzed on the INCAT total score using the RSDS. An overview of all other planned secondary analyses is provided in Table 2.

**Table 2:** Analyses performed at each study period for secondary outcomes, IgG levels, and health-related quality of life variables

	Period (analysis set)		
	IVIg restabilization (PSDS)	SC treatment (ITTS, PPS)	IVIg rescue (RSDS)
INCAT			
Total	Overall: - Descriptive statistics - Change from reference visit - Time to first improvement on IgPro10: Kaplan-Meier estimates	- By treatment and by treatment and subgroup: - Descriptive statistics - Comparison between treatments of changes from reference visit: exact Jonckheere-Terpstra test^a^ - Comparisons between each IgPro20 dose group and placebo group, and the comparison between the two IgPro20 dose groups of changes from reference visit: Wilcoxon rank sum test^b^ - Time to CIDP relapse or withdrawal for any other reason: Kaplan-Meier estimates^c^	Overall: - Descriptive statistics - Change from reference visit - Time to improvement after CIDP relapse with IgPro10 rescue: Kaplan-Meier estimates
Mean grip strength (Dominant/non-dominant hand), MRC sum score, I-RODS			
	Overall: - Descriptive statistics - Change from reference visit - Time to first improvement on IgPro10: Kaplan-Meier estimates	By treatment and by treatment and subgroup: - Descriptive statistics - Comparison between treatments of changes from reference visit; exact Jonckheere-Terpstra test^a^ - Comparisons between each IgPro20 dose group and placebo group, and the comparison between the two IgPro20 dose groups of changes from reference visit; Wilcoxon rank sum test^b^	Overall: - Descriptive statistics - Change from reference visit
Electrophysiological parameters (average distal latency, average proximal latency, overall average conduction velocity, average conduction block, and average compound muscle action potential amplitude)			
		By treatment and by treatment and subgroup: - Descriptive statistics - Change from reference visit - Between-treatment comparisons of the changes from baseline: analysis of variance (ANOVA) model^d^	
IgG level			
	Overall: - Descriptive statistics - Change from reference visit	By treatment and by treatment and subgroup: - Descriptive statistics - Change from reference visit	Overall: - Descriptive statistics - Change from reference visit
EQ-5D dimensions, TSQM, WPAI-GH			
	Overall: - Descriptive statistics	By treatment: - Descriptive statistics - Change from reference visit - Comparison between treatments of changes from reference visit; exact Jonckheere-Terpstra test^a^ - Comparisons between each IgPro20 dose group and placebo group, and the comparison between the two IgPro20 dose groups of changes from reference visit; Wilcoxon rank sum test^b^	Overall: - Descriptive statistics
Patient preference for treatment questionnaire			
		By treatment: - Descriptive statistics	

*EQ-5D* EuroQoL 5-Dimension Questionnaire, *IgG* immunoglobulin G, *ITTS* intention-to-treat set, *IVIg* intravenous immunoglobulin, *MRC* Medical Research Council, *PSDS* pre-randomization safety data set, I-*RODS* Inflammatory-Rasch-built Overall Disability Scale, RSDS, rescue safety data set, *SC* subcutaneous, *TSQM* Treatment Satisfaction Questionnaire for Medication, *WPAI-GH* Work Productivity and Activity Impairment Questionnaire for General Health

^a^Exact Jonckheere-Terpstra test see ref [54]

^b^For each pair-wise comparison, the one-sided *p* value from the Wilcoxon rank sum test and the Hodges-Lehmann estimate of the median difference between treatments will be presented together with the corresponding two-sided 95 % Moses confidence interval

^c^Overall between-treatment comparison will be performed using the log-rank test for trend, all pair-wise comparisons will be performed using the log-rank test

^d^Treatment and region (Japan, non-Japan) will be used as explanatory variables. Within the framework of this ANOVA model, comparisons of each IgPro20 dose with placebo, and the comparison of the two IgPro20 doses will be performed. For these comparisons, the least squares mean for each treatment group, an estimate of the difference between treatments (if applicable), corresponding 95 % confidence interval and 2-sided p-values will be presented

**Table 3:** Overview of sensitivity analyses

Withdrawal reasons	Assignment of patients in:				
	Primary analysis	Sensitivity analysis			
		A	B	C	D^a^
The patient experiences a CIDP relapse during the SC treatment period (lack of efficacy)	Relapser	Relapser	Relapser	Relapser	Relapser
The investigator advises that the patient’s safety or wellbeing could be compromised by further participation in the study (physician decision)	Relapser	Non-relapsers	Relapser	Not used for analysis	Censored
The patient receives prohibited medication (protocol violation)					Censored
Other withdrawal reason (other, adverse event, death, lost to follow-up, protocol violation, study termination by sponsor, and withdrawal by patient)			Non-relapsers		Censored
Patient continues to study end	Non-relapsers	Non-relapsers	Non-relapsers	Non-relapsers	Non-relapsers

*CIPD* chronic inflammatory demyelinating polyneuropathy, *SC* subcutaneous

^a^Exploratory sensitivity analysis

#### Safety evaluation

Duration of exposure, overall exposure in patient years, the total dose of IgPro10 or IgPro20 received, and the number of infusions will be summarized for the three separate study periods, using descriptive statistics. Analyses of all AEs, including deaths, serious AEs, other significant AEs, and AEs leading to withdrawal of study drug or to study discontinuation will be conducted on both a patient level and an infusion level and will be summarized for the three separate study periods, using descriptive statistics. For SC infusions, each infusion session will be counted separately. IV infusions administered over more than 1 day will be counted as separate infusions. The time to onset and duration of the most frequent AEs will be summarized using descriptive statistics. Continuous clinical laboratory parameters (hematology and serum chemistry) will be summarized for the IVIg restabilization period, by visit, using descriptive statistics. Likewise, values and changes will be summarized for the SCIg treatment (by treatment) and IVIg rescue period.

#### Sensitivity analyses

Sensitivity analyses will be performed with modified primary endpoint definitions to investigate the potential bias due to the inclusion of dropouts without CIDP relapse (patients who withdraw from the study due to any reason other than CIDP relapse) in the primary endpoint. For each sensitivity analysis, the analysis performed for the primary endpoint will be repeated, including the exact Cochran-Armitage trend test, one-sided Fisher’s exact tests for the pair-wise treatment comparisons and all estimates and confidence intervals.

For the primary analysis, only patients who complete the study without recording a relapse are considered nonrelapsers. All other patients, including those who withdraw for any reason other than relapse, are considered relapsers. Four sensitivity analyses will be performed that assign a different status’ (relapse or nonrelapser) to patients who are withdrawn from the study for reasons other than efficacy (Table 3):

**Table 4:** Subgroup analyses by endpoint

Subgroup	Primary efficacy analysis of Relapse rate	INCAT total score	Mean grip strength (dominant/ nondominant hand)	I-RODS	MRC sum score	Time to relapse or withdrawal for any other reason	IgG serum levels
Sex (male/female)	X	X	X	X	X	X	
Age group (≥18 years to ≤ 65 years, and > 65 years)	X	X	X	X	X	X	
Prestudy IVIg treatment modality (IVIg maintenance therapy, acute IVIg therapy)^a^	X	X	X	X	X	X	
Region (Japan/non-Japan)	X						X
Relapse status (yes/no)							X
IVIg Dependency criterion: I-RODS or grip strength	X^b^						

*IgG* immunoglobulin G, *INCAT* Inflammatory Neuropathy Cause and Treatment, *IVIg* intravenous immunoglobulin, *MRC* Medical Research Council, *I-RODS* Inflammatory-Rasch-built Overall Disability Scale

^a^For Japanese subjects only

^b^This subgroup analysis will only be conducted if the group size is ≥ 30

- Sensitivity analysis A, withdrawn patients are considered nonrelapsers.
- Sensitivity analysis B, withdrawn patients are considered according to withdrawal reason.
- Sensitivity analysis C, withdrawn patients are not included in the analysis.
- Sensitivity analysis D (exploratory), withdrawn patients will be censored at the date of their last visit. Kaplan-Meier estimates of the probability of having a relapse at 24 weeks will be calculated. Between-treatment comparison will be performed using the log-rank test for trend. When an overall trend is demonstrated, two subsequent one-sided comparisons will be performed using the regular log-rank test.

#### Examination of subgroups

Exploratory subgroup analyses will be performed for endpoints in the SC treatment period (Table 4). The subgroup analysis for age group will only be conducted if the size of the smallest group exceeds 10 patients. The percentage of patients experiencing a CIDP relapse (or withdrawing for any other reason) during the SC treatment period within each subgroup category will be presented by treatment group.

#### Interim analyses

The independent data monitoring committee will be unblinded for a formal interim analysis based on the outcome data of at least 60 patients at SC week 12. The decision will be based on the outcome at 12 weeks in the SC treatment period rather than on the outcomes. The decision following the interim analysis will be to either stop the study for futility or to go on as planned and stop with the originally planned sample size.

### Study protocol amendments

During the conduct of this trial, the protocol has been amended five times. Amendments 1 and 2 (17 Nov 2011 and 10 Dec 2012) addressed only local changes and Amendment 5 (15 Sep 2015) was mainly to update safety information. With Amendment 3 and 4 (12 Apr 2013 and 11 Sep 2014), the following two important changes were implemented:

(1) The IVIg withdrawal phase was modified to an IgG dependency test and additional deterioration criteria were implemented as described above. Fulfillment on one of these criteria, in the event of an unchanged INCAT score, qualified the patient to move to the next study phase (Amendment 3). Relapse rates in the IgPro20 groups were anticipated to increase after this change due to the fact that significant decrease in grip strength (i.e., 8-point deterioration) is not always accompanied by a corresponding worsening in adjusted INCAT score by 1 point [40]. To correct for the new assumptions for relapse percentages underlying the power calculation, the sample size was increased from 150 to 174, and the screening numbers were increased from 250 to 350. The underlying assumptions were that 90 % of subjects would be recruited after Amendment 3 and the dropout rates for placebo subjects would increase to 15 % (while being around 10 % in the active treatment groups).

(2) The length of time required for prestudy IVIg has been reduced to 8 weeks (Amendment 4). The change in this requirement is not expected to adversely affect the outcomes of the SC treatment period because all patients must show IgG dependency (up to 12 weeks) and IVIg restabilization (up to 13 weeks) before randomization and start of SC treatment.

### Data management and auditing

Data were entered directly into the Medidata Rave ® eCRF by study sites. eCRF access for data entry was only given to site personnel. Data generated throughout the study were monitored, and the eCRFs were checked against the subject records for completeness and accuracy. This function was completed by a CRO with defined delegated responsibility.

Following completion of the eCRFs, the data were checked electronically for consistency and plausibility. Queries were generated for questionable data. All queries had to be resolved in a timely manner by the investigator. For this purpose, data cleaning status reports have been generated on an ongoing basis. In addition, the reconciliation of all external data against the eCRF took place to assure data consistency between the systems. On top of this, selected tables, figures, and listings (TFLs) were generated by Chiltern Stats for clean data review. Three clean data slices were scheduled according to the enrolment rate.

All study data, irrespective of the medium in which they are stored, will be handled in strictest confidence in accordance with applicable data protection laws: e.g., the European Data Protection Directive [95/46/EC] and the US Health Insurance Portability and Accountability Act [HIPAA]), and will be evaluated by the sponsor and/or a competent regulatory authority in an anonymized form.

## Discussion

### Design

To ensure that the true treatment difference of IgPro20 compared to placebo can be determined, the present study includes three separate periods. First, an IgG dependency test period is included to ensure that only patients are treated who are still in need of IgG. The necessity of including a run-in period in which the IVIg dose is reduced or withheld to prove IgG dependency has become clear during the RMC-trial [41]. Next is the IVIg restabilization period, which will be performed with the 10 % IVIg product IgPro10 to ensure standardized IVIg restabilization conditions before initiation of placebo-controlled, randomized, SC treatment with IgPro20 or placebo.

Finally, eligible patients will be randomized to show that IgPro20 can maintain the improvement in INCAT score achieved during the IVIg restabilization period in patients with CIDP.

### Selected doses

The IVIg loading dose and maintenance doses every 3 weeks for the restabilization period is based on the EFNS/PNS Guidelines and evidence from a large international study [26, 32]. For the SC treatment period, an equal activity of SCIg and IVIg is assumed. Furthermore, several smaller studies in CIDP and in MMN suggest efficacy of SCIg with dosages from 0.09 to approximately 4 g/kg bw [15, 16, 22, 42]. Therefore, two doses are being tested in this study: a lower dose with putative acceptable efficacy and a dose significantly above the lower SCIg dose and above the weekly equivalent of the recommended IVIg dose (0.33 g/kg bw) but still within acceptable volumes to be infused at weekly intervals.

### Appropriateness of measurements

For the diagnosis of CIDP in this study, the most recent guideline from the EFNS/PNS society will be used [26]. These criteria are well accepted in the neurological community for the diagnosis of CIDP. Well-established and accepted outcome measures are used to assess limb disability (INCAT score), muscle strength (MRC sum scores), and tests of actual grip strength. INCAT and MRC sum scores have been widely used in other studies and publications (INCAT [27, 43, 44]; MRC score [32, 45–48]). The adjusted INCAT score is applied throughout the study because changes in the function of the upper limbs from 0 (normal) to 1 (minor symptoms) or from 1 to 0 are not considered by regulatory agencies to be clinically significant in all patients [32].

Grip strength has been found to be significantly associated with arm disability values over time, implying that grip strength can be applied as an index of arm function recovery in patients with immune-mediated polyneuropathies [29, 40, 49]. The I-RODS is currently the only linearly weighted activity and social participation limitation scale. The I-RODS captures a very broad range of difficulty items and has successfully been validated against the Overall Disability Sum Score with an intraclass correlation coefficient of 0.85 and a very high test reliability of repeated measurements with an intraclass correlation coefficient of 0.97–0.99 [28]. The I-RODS responsiveness in CIDP has recently been demonstrated [50]. Nerve conduction studies provide objective and reliable indices of the integrity and function of peripheral nerves independent of patient cooperation. They are often included in clinical studies that evaluate treatments for peripheral neuropathy to assess and/or confirm the efficacy of treatment [51–53]. In the situation of IgG withdrawal, electrophysiological parameters have not been determined before. All quality of life measures (EQ-5D, TSQM, and WPAI) have been extensively validated in numerous therapeutic areas and have been shown to be robust based on sensitivity, reliability, and internal consistency [39].

For the comparison of different treatments, an assessment of the patient’s preference, which will be done with a Patient Preference for Treatment Questionnaire, is important. The safety measures used in this study (AEs, vital signs, physical and neurological examinations, laboratory investigations, and viral safety) are routine procedures for clinical studies. All other efficacy and safety measurements used in the current study are also widely used and generally recognized in the medical literature as relevant in the clinical evaluation of Ig therapy.

As the study involved many centers throughout the world, special attention was given to the standardization of outcome measures. Investigator meetings with dedicated training, on-site training, and web-based trainings were all mandatory for investigators. Central eligibility checks and strict monitoring protocols were added to the standardization. All patient questionnaires were provided in the local language of the specific country and were validated for that language.

The findings of this trial will increase knowledge about the use of SCIg in the maintenance management of CIDP patients and add another treatment modality to the armamentarium of neurologists to choose from when treating these patients. The expected benefits of weekly SCIg are a reduction in systemic AEs and an increase in patient autonomy and quality of life through self-treatment. High IgG peak levels and low trough levels are avoided, and a more constant IgG level is achieved. This is expected to result in a reduced wear-off effect at the end of the dosing period.

### Trial status

The trial started in March 2012 and completed patient recruiting by the end of November 2015. Currently, 289 patients have been screened, and 172 have been randomized. Study completion is expected at the end of 2016.

All items from the World Health Organization Trial Registration Data Set can be found at http://apps.who.int/trialsearch/Trial2.aspx?TrialID=EUCTR2011-003448-28-DE.

## Abbreviations

AE, adverse event; AESI, adverse events of special interest; ANOVA, analysis of variance; BW, body weight; CIDP, chronic inflammatory demyelinating polyneuropathy; EFNS/PNS, European Federation of Neurological Societies/Peripheral Nerve Society; EQ-5D, EuroQoL 5-Dimension Questionnaire; HRQL, health-related quality of life; INCAT, inflammatory, neuropathy, cause and treatment; I-RODS, Inflammatory-Rasch-built Overall Disability Scale; ITTS, intention-to-treat set; IVIg, intravenous immunoglobulin; MRC, Medical Research Council; PATH, Polyneuropathy and Treatment with Hizentra; PPS, per-protocol set; PSDS, pre-randomization safety data set; RSDS, rescue safety data set; SC, subcutaneous; SCIg, subcutaneous immunoglobulin; SDS, safety data set; TEAE, TREATMENT-emergent adverse event; TSQM, Treatment Satisfaction Questionnaire for Medication; ULN, upper limit of normal; VAS, visual analog scale; WPAI-GH, Work Productivity and Activity Impairment Questionnaire for General Health

## Appendix

### List of all ethical committees that approved the study

#### Australia

St. Vincent’s Hospital Melbourne Human Research Ethics Committee, Research Governance Unit

Level 5, Mary Aikenhead Building

27 Victoria Parade

Fitzroy, Victoria 3065, Australia

#### Belgium

Commissie Medische Ethiek van Universitaire Ziekenhuizen K.U. Leuven

Campus Gasthuisberg E330

Herestraat 49

Leuven 3000, Belgium

#### Canada

IRB Services

372 Hollandview Trail, Suite 300

Aurora, Ontario L4G 0A5, Canada

University Health Network Research Ethics Board

700 University Avenue, 10/F, Room 10-56

Toronto, Ontario M5G 1Z5, Canada

#### Czech Republic

Eticka komise Fakultni nemocnice Hradec Kralove

Sokolska 581, Hradec Kralove 500 05,

Czech Republic

#### Estonia

Tallinn Medical Research Ethics Committee, Institute of Health Department

Hiiu 42

Tallinn 11619, Estonia

#### Finland

Nathional Committee on Medical Research Ethics TUKUA

Lintuladenkuja 4

Helsinki FI-00530, Finland

#### France

CPP Sud-Ouest et Outre Mer III

Service de Pharmacologie Clinique, Bat. 1A

Hôpital PELLEGRIN

Place Amélie Raba Leon

Bordeaux Cedex 33076, France

#### Germany

Ethikkommission der Medizinischen Fakultät der Ruhr-Universität Bochum

BG-Universitätskliniken Bergmannsheil

Bürkle-de-la-Camp-Platz 1

Bochum 44789, Nordrhein-Westfalen, Germany

#### Israel

Helsinki Committee of Tel Aviv Sourasky Medical Center

Tel Aviv Sourasky Medical Center, 6 Weizmann Street

Tel-Aviv 64239, Israel

Helsinki Committee

Rambam Health Care Campus

8 Ha’alia st., Bat Galim

Haifa 31096, Israel

#### Italy

Comitato Etico Dell’Universitá Degli Studi G. D’Annunzio E Della ASL 2 Lanciano-Vasto-Cheiti

Via dei Vestini, 31

Chieti Scalo 66100, Italy

Comitato Etico Dell’Azienda Ospedaliera S. Andrea Di Roma

Via di Grottarossa 1035-1039 NAP

Rome 00189, Italy

Comitato Etico dell’Azienda Ospedaliera Univeritaria S. Martino di Genova

Largo Rosanna Benzi, 10 NAP

Genova 16132, Italy

Comitato Etico Dell’ Azienda Ospedaliera Universitaria S. Giovanni Battista Di Torino

Corso Bramante 88/90

Torino 10126, Italy

#### Japan

Institution Review Board, Tokyo Medical and Dental University, University Hospital of Medicine

1-5-45, Yushima

Bunkyo-ku, Tokyo 113-8519, Japan

Institution Review Board, Yamaguchi University Hospital

1-1-1, Minamikogushi

Ube, Yamaguchi 755-8505, Japan

Institutional Review Board, National Defense Medical Hospital

3-2 Namiki

Tokorozawa-shi, Saitama 359-8513, Japan

Institutional Review Board, Chiba University Hospital

1-8-1, Inohana, Chuo-ku

Chiba-shi, Chiba 2608677, Japan

Instititutional Review Board, Nagoya University Hospital

65, Tsurumaicho, Showa-ku

Nagoya, Aichi 466-8560, Japan

Institutional Review Board, Aomori Prefectural Central Hospital

2-1-1 Higashi Tsukurimichi

Aomori 030-8553, Japan

Institutional Review Board of Kitasato University Hospital

1-15-1 Kitasato, Minami-ku

Sagamihara-shi, Kanagawa 252-0375, Japan

Institutional Review Board, Juntendo University Hospital

Hongo 3-1-3

Bunkyo-ku, Tokyo 113-8431, Japan

#### Netherlands

Academic Medical Center, University of Amsterdam

Medisch Ethische Toetsingscommisie

E2-170

Meibergdreef 9

Amsterdam 1105 AZ, The Netherlands

#### Poland

Komisja Bioetyczna przy Uniwesytecie Medycznym w Lublinie

Al. Racklawickie 1

Lublin 20-059, Poland

#### Spain

Agencia de Ensayos ClÍnicos

Hospital Universitario Vall d’Hebron

Edifici Institut de Recerca, 3 ͣ planta

Passeig Vall d’Hebron, 119-129,

08035 Barcelona, Spain

#### United Kingdom

NRES Committee South Central

Room 002, TEDCO Business Centre

Rolling Mill Road

Jarrow NE32 3DT, United Kingdom,

South Central - Oxford C

South West REC Centre

Level 3, Block B, Whitefriars, Lewins Mead

Bristol BS1 2NT, United Kingdom

#### United States

Western Institutional Review Board

1019 39th Ave. SE Suite 120

Puyallup, WA 98374, USA

Northwestern University Institutional Review Board

Arthur Rubloff Building, 7th floor

750 N. Lake Shore Drive

Chicago, IL 60611, USA

Houston Methodist Research Institute - Institutional Review Board (new)

6565 Fannin St. MGJ6-014

Houston, TX 77030, USA

Human Subjects Committee, University of Kansas Medical Center

3901 Rainbow Boulevard

Kansas City, KS 66160

Health Sciences Institutional Review Board

Suite 4700

1200 North State Street

Los Angeles, CA 90033, USA

St. Joseph’s Hospital and Medical Center

Institutional Review Board for Human Research

350 West Thomas Road

Phoenix, AZ 85013, USA

Office of Research and Sponsored Programs

Weill Cornell Medical College

1300 York Ave, Box 5,

New York, NY 10065, USA

University of Miami Human Subjects Research Office

1500 NW 12th Avenue, Suite 1002

Jackson Medical Towers East

Miami, FL 33136, USA

Human Research Protection Program, University of Minnesota

D528 Mayo Memorial Building

420 Delaware St. SE, MMC 820

Minneapolis, MN 55455, USA

Duke University Health System Institutional Review Board

2424 Erwin Road, Suite 405

Campus Box 2712

Durham, NC 277005, USA
